# PEER (DYADIC) SUPPORT: A FEASIBILITY STUDY FOR OLDER AFRICAN AMERICAN WOMEN WITH HYPERTENSION

**DOI:** 10.1093/geroni/igad104.2914

**Published:** 2023-12-21

**Authors:** Angela Groves, Maria Roche-Dean, Emily Bosak

**Affiliations:** Western Michigan University, Portage, Michigan, United States; Western Michigan University, Kalamazoo, Michigan, United States; Western Michigan University, Kalamazoo, Michigan, United States

## Abstract

African American women have a higher prevalence of hypertension than women of other ethnicities. Interventions have not adequately addressed the higher prevalence of hypertension in this population. Social support networks can be an effective strategy for improving hypertension self-management behaviors. The objective of this study was to evaluate the feasibility of an 8-week peer (dyadic) support intervention to improve diet adherence and reduce systolic blood pressure among older African American women with hypertension and describe the relationship that occurred during the intervention. This mixed methods study used a convergent parallel design. A total of 26 African American women diagnosed with hypertension, aged 60 and older were paired to form 13 dyads. All participants completed a 3-hour training session consisting of home blood pressure monitoring, DASH diet, and communication. Participants completed pre and post DASH diet surveys, and the Social Support Survey (MOS) post intervention. At the end of the 8-week intervention, participants discussed their experiences in a 60-minute focus group session. Content analysis was used to analyze focus group interviews. Preliminary data analysis will be presented. Emerging themes included, accountability, epiphany, challenges with hypertension management, communication with each other, and receiving support. Preliminary findings suggest that peer (dyadic) support is feasible and can be an effective intervention strategy to improve diet and blood pressure management. Future studies should address specific challenges to hypertension management identified by participants. Lastly, this study used random within site pairing for participants without an identified partner. Future studies should develop criteria for pairing partners.

